# The impact of payer status on hospital admissions: evidence from an academic medical center

**DOI:** 10.1186/s12913-021-06886-3

**Published:** 2021-09-07

**Authors:** Yanying Zhao, Ioannis Ch. Paschalidis, Jianqiang Hu

**Affiliations:** 1 School of Management, Fudan University, 670 Guoshun Road, Yangpu District, Shanghai, 200433 China; 2 Departments of Electrical & Computer Engineering, Systems Engineering, and Biomedical Engineering, Boston University, 8 St Marys Street, Boston, Massachusetts, 02215 USA

**Keywords:** Payer status, Hospitalization, Low-income, Uninsured

## Abstract

**Background:**

There are plenty of studies investigating the disparity of payer status in accessing to care. However, most studies are either disease-specific or cohort-specific. Quantifying the disparity from the level of facility through a large controlled study are rare. This study aims to examine how the payer status affects patient hospitalization from the perspective of a facility.

**Methods:**

We extracted all patients with visiting record in a medical center between 5/1/2009-4/30/2014, and then linked the outpatient and inpatient records three year before target admission time to patients. We conduct a retrospective observational study using a conditional logistic regression methodology. To control the illness of patients with different diseases in training the model, we construct a three-dimension variable with data stratification technology. The model is validated on a dataset distinct from the one used for training.

**Results:**

Patients covered by private insurance or uninsured are less likely to be hospitalized than patients insured by government. For uninsured patients, inequity in access to hospitalization is observed. The value of standardized coefficients indicates that government-sponsored insurance has the greatest impact on improving patients’ hospitalization.

**Conclusion:**

Attention is needed on improving the access to care for uninsured patients. Also, basic preventive care services should be enhanced, especially for people insured by government. The findings can serve as a baseline from which to measure the anticipated effect of measures to reduce disparity of payer status in hospitalization.

**Supplementary Information:**

The online version contains supplementary material available at (10.1186/s12913-021-06886-3).

## Background

Based on their primary insurance payers, patients can be classified into five groups: Medicare, Medicaid, Commercial, Uninsured, and Other, among which Medicare and Medicaid are government administered programs whereas commercial insurance is provided by private insurers. Medicare is available for patients 65 or older, younger people with disabilities, and dialysis patients, and Medicaid is available for low-income individuals or pays for costs as a supplement to Medicare. In reality, health care service is not experience equity by all populations. For example, there are 49.6% of adults ages from 16 to 64 met difficulties in access to care in Massachusetts due to insurance provider issues in 2015 [1]. Therefore, reducing inequity has always been the focus of study and health care reform in U.S.

Generally, equity in healthcare encompasses timely access, equivalence of care, and absence of avoidable or remediable differences among groups of people, pertinent to distinct social, economic, demographical or geographical criteria[2]. Previous studies have demonstrated that inequity exists in the entire process of care, including access [3–5], in-hospital experience [6], treatment and outcomes [7–9]. In terms of access to care, for example, Hsiang et al. [4] found that Medicaid patients are faced with 1.6-fold lower likelihood in scheduling a primary care appointment and 3.3-fold lower likelihood in specialty appointment comparing to private insured patients. The avoidable hospitalization rate for Medicaid and uninsured patients is higher than privately insured patients [10]. These studies indicate that more attention should be given to low-income patients to improve their access to care.

Expanding insurance coverage is one of the actions taken by the government to improve patients’ equity in health care. For example, Massachusetts issued a bill titled “An act providing access to affordable, quality, accountable health care” in 2006, which increased the insurance coverage proportion in non-elderly adults (ages from 16 to 64) from 86.0% in 2006 to 95.7% in MA [1]. Generally speaking, health insurance coverage expansion has improved patients access to care [11, 12]. Dzordzormenyoh [13] further investigated the impact of Medicaid expansion from three aspects: access to a physician, access to basic healthcare, and access to specialized care, and finds that Medicaid expansion can significantly improve the access to basic care and specialized care, but access to physicians is weakened due to the low reimbursement rate and complex paper work. Decker et al. [14] found that 31% office-based physicians are unwilling to accept any new Medicaid patients, while only 17% office-based physicians are unwilling to accept any new privately insured patients. The limitation access to office-based physicians drives Medicaid patients to seek care from public institutions, for example, hospital emergency departments and outpatient departments [15, 16]. According to Sutton et al. [17], 40% of inpatients in safety-net hospitals were either covered by Medicaid (34.7%) or uninsured (6.7%), compared to just 20.7% of inpatients in non-safety-net hospitals (16.8% Medicaid and 3.9% uninsured).

Medicaid is a costly program for government to sustain. Faced with the increasing Medicaid patients, the finance problem in public institution becomes more sever. How to obtain sustainable and secure finance support becomes the primary issue for their administrators [18]. Some studies claim that the improvement on access to care by Medicaid expansion would be weakened in the long run for public institutions which may have difficulty in giving continued and quality care [19]. It is obvious that in addition to insurance coverage expansion, more measures should be taken to protect low-income patients’ health.

Therefore, it is very important for policy makers or hospital administrators to have a comprehensive understanding on the disparity of payer status in access to care. However, most of related studies are either disease-specific or cohort-specific [3, 5, 7, 8]. Few quantify the impact of payer status on access to care, especially for hospitalization, with a large controlled study from a more “macro” level. To our knowledge, Cai et al. [20] is one of the few studies exploring the differences in hospitalization between Medicaid patients and private-pay patients within a facility. Studying from the level of a facility is conducive to find the inherent difference in hospitalization, and thus promote hospitals to find reasons and to take actions correspondingly [20].

Following the spirit of Cai et al. [20], this study aims to quantify the disparity of payer status in hospitalization from the perspective of a facility. Comparing to Cai et al. [20], our study includes all payer status, and compares their impact on hospitalization based on the value of standard regression coefficients. It is helpful not only for promoting a more comprehensive understanding on the impact of payer status on hospitalization, but also for providing hospital administrators with a basis for deciding which group of patients should be given more attention.

It is worth mentioning that this study is conducted based on a safety-net hospital, which is required to provide health care to all patients regardless of their ability to pay [21]. Ideally, the hospitalization rate should be the same for patients with the same health conditions, and any disparity in hospitalization should be solely due to patients severity of illness. Given the nature of a safety-net hospital, we think it can provide us with a more appropriate condition to reveal the inherent gaps of hospitalization among different payer status.

## Methods

### Data

The data we used come from a large academic medical center in the U.S. Only patients who had visit records during the period of 5/1/2009-4/30/2014 were included in our study, resulting in a dataset containing information for 490,761 patients. For each patient in this set, we extracted their demographic characteristics and medical history (outpatient/emergency room visit records, diagnoses, medications, hospitalizations) during the period of 5/1/2006-4/30/2014. Table 1 describes the extracted features corresponding to each patient.

**Table 1:** Features extracted for each patient

Ontology	Number of factors	Examples
Demongraphics	6	Sex; Age; Race; Marital Status; Language; Zip-Code
Payer	5	Government-Sponsored (Medicare, Medicaid, Health safety net, commonwealth care); Private-sponsored (Commercial, Accident); Uninsured(Self-pay, Unknown); Other; Multiple-sponsor(Insured by both government and private sponsors)
Primary Diagnose	22	e.g., Infections (ICD9: 001-139); Neoplasms (ICD9: 140-239); Endocrine (ICD9: 240-259); Nutrition (ICD9: 260-269); Blood (ICD9: 280-289); Circulatory (ICD9: 390-459); Injury&Position (ICD9: 800-999); Illdefined (ICD9: 780-799); Supplementary 1 (ICD9: V01-V91)
Chronic Disease	1	e.g., Hypertensive disease (ICD9: 401.0-401.99); Diabetes mellitus (ICD9: 250.00-250.13, 250.22, 250.40-250.93); HIV (ICD9: 042.0-043.9, 079.53, v65.44); etc.
Prescription	1	Whether there are prescriptions or not
Sevice by department	3	Inpatient, Outpatient, Emergency Room
Admissions	22	The cause of hospitalization.

^1^ICD9 is a commonly used medical coding system for disease. See website [32] for full list of diseases.

^2^Supplementary 1 is a disease type in ICD9, defined as Supplementary classification of factors influencing health status and contact with health services

### Dependent Variable

A binary variable *Admission* is defined indicating whether a patient has been hospitalized during the entire 5 year period we considered.

**Fig. 1:**
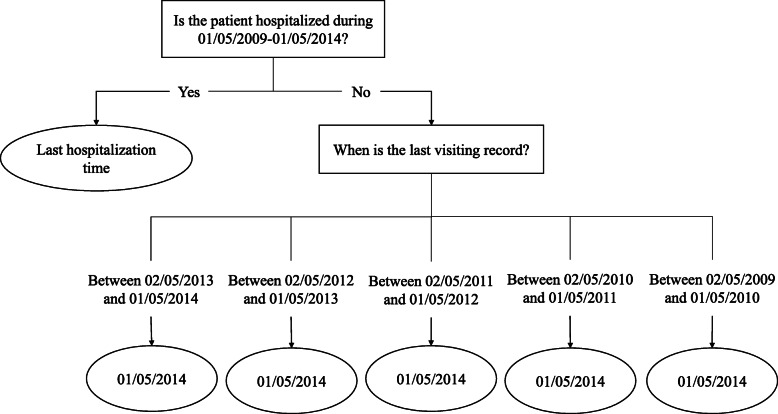
The process of obtaining a patient’s target time

We find there exists significant imbalance between the sample size of the admitted and non-admitted patients. In order to reduce the imbalance, we introduce a “floating” time node – “target time”, which is similar to the “target year” used in Brisimi et al. [22]. The value of target time is defined according to the following rules: (1) For patients who have been hospitalized during the 5-year period, we define the last hospitalization time as their target time. (2) For patients who have not been hospitalized during the 5-year period, we define the last day of the sub-period (the length of a sub-period is 365 or 366 days) in which their last visiting record falls as their target year. Figure 1 present the process of obtaining a patient’s target time. The model we will derive seeks to predict admission at the target time. With this method, the number of patients labeled as admitted accounts for 16.53% of the total number of patients.

### Independent Variable

There are 8 payers in our sample: Medicare, Medicaid, Commonwealth care (offered by the state), Health safety net, Accident, Commercial, Self-pay and Other. According to the nature of payers, we classified them into 5 groups: government-private-sponsored, government-sponsored, private-sponsored, uninsured, and other (see Appendix A in Additional file 1 for detailed information on group composition). We use a categorical variable *Payer* to denote a patient’s payer status.

We note that some patients have multiple payers during the specific time-period. Since the insurance status of a patient may change over time, we consider the latest status as their payer status.

### Covariates

In order to organize all the available information in a uniform way for all patients, some preprocessing of the data is needed. Pre-processing steps include imputing missing values and summarizing historical factors.

#### Sample imputation

Missing data imputation seeks to replace missing data with substituted values. We note that some records lack the information on the primary diagnosis. We use a matching algorithm to impute the missing values based on the following rules: (1) for patients who have prescriptions, we select the most common disease treated with their prescriptions as the substituted value; (2) for patients who have no prescriptions, we select the most common disease according to the history of their medical visits; and (3) for patients who have neither prescriptions nor prior medical visits, we label their diagnosis as “Unknown.” The most common disease here means the disease with the highest frequency. For example,if a patient visit the medical center 3 times due to the disease of Circulatory, Circulatory and Digestive, respectively, her most common disease is labeled as Circulatory.

We note that a patient may seek care for different causes that correspond to different primary diagnoses in their visiting records. In order to keep each patient’s primary diagnosis unique in our sample, we choose the most common visit (or hospitalization) cause as the primary diagnosis for a patient who has never (ever) been hospitalized. We define *Diagnosis* as an unordered categorical variable denoting a patient’s primary diagnosis (23 categories; cf. Table 2).

**Table 2:** Descriptive Characteristics

Variables	*Admissions*^1^		*P*-value^2^
	=1	=0	
*Sex*			0.000
Female	48.1	51.9	-
*Race*			0.000
Asian	2.77	5.25	-
Hispanic	19.1	19.1	-
Other Race	5.08	7.69	-
Unknown Race	0.7	7.11	-
White	37.4	33.1	-
*Marital*			0.000
Divorced	5.75	2.72	-
Other Marital	1.92	5.05	-
Seperated	2.53	1.36	-
Single	56.1	65.9	-
Unknown Marital	0.02	0.13	-
Widow	5.47	1.58	-
*PrscNum*	0.52	0.39	0.000
*Chronic*	19.7	80.3	0.000
*Combinations*	6.65	2.18	0.000
*Diagnosis*			0.000
Blood	0.96	0.36	-
Circulatory	13.3	4.12	-
Congential Anomalies	0.21	0.32	-
Digestive	9.7	6.97	-
Endocrine	2.33	3.04	-
Genitourinary	4.72	5.45	-
Illdefined	4.38	12.2	-
Infections	4.02	2.94	-
Injury & Poison	13.1	8.92	-
Mental	1.64	6.56	-
Metabolic & Immune	1.35	1.38	-
Musculoskeletal	5.81	10.77	-
Neoplasms	6.37	2.94	-
Nutrition	2.68	7.73	-
Pregnancy	16.0	1.44	-
Respiratory	6.18	3.42	-
Secondary	1.33	0.50	-
Skin	2.75	4.64	-
Supplementary 1	1.9	13.0	-
Unknown Diagnosis	0.08	3.13	-
*Payer*			0.000
Multiple payers	8.24	5.08	-
Government	65.7	50.8	-
Private	23.1	35.0	-
Other Payers	1.51	3.70	-
Uninsured	1.44	5.44	-
*ERVisits*			0.000
=0 ER visit record	86.2	73.9	-
=1 ER visit record	8.22	19.6	-
>1 ER visit records	5.61	6.48	-
*PriorAdmissions*			0.000
=0 prior admissions	66.6	99.3	-
=1 prior admissions	16.6	0.53	-
>1 prior admissions	16.7	0.15	-

^1^The 3rd and 4th column report the percentage of admitted and non-admitted patients, respectively, with the given feature for categorical variables or the mean over admitted patients for continuous features.

^2^A Chi-square test is used to check whether the difference of proportions (percentages) among different categories of a categorical variable is significant. A t-test is used to check the null hypothesis that means of continuous variables among different categories are equal. The *p*-values are presented in the 5th column

#### Summarization of historical factors

We summarize records three years before a patient’s target time. We assigned a patient’s most common primary diagnosis in the variable we call *Diagnosis*. In order to include other diagnoses, we introduce a variable called *Combinations*, indicating the total number of diseases of a patient except her *Diagnosis*. *PrscNum* denotes the total number of prescription order in the record for this patient that are attributed to the primary diagnosis, which takes values in the interval 0 to 30, with 30 including cases of 30 or more prescriptions. *Prior* *Admissions* and *ER* *Visits* are both ordered categorical variables coded 0 to 2, with 0 representing no earlier visit/admission records, 1 representing 1 earlier visit/admission records, and 2 representing more than 1 earlier visit/admission records. *Chronic* is a binary indicator variable indicating whether a patient’s disease is chronic.

We also include other demographic factors: *Age*, *Race*, *Marital* *Status*. *Age* is an ordinal category variable, coded 1 to 8, with 1 indicating ages no more than 10 years, 2 indicating ages from 10 to 20 years, etc., up to 7 corresponding to ages from 60 to 70 years, and 8 indicating ages more than 70 years. We convert categorical features–including *Race*, *MaritalStatus*, *Diagnosis* and *Payer* –into a set of binary variables with one-hot encoding. For all binary variables, zero indicates lack of this feature. In total, there are about 50 features for each patient. We present a summary description of the variables in Appendix D in Additional file 1.

#### Patient and feature selection

We remove features which are only available for a small number of patients. Patients who are under 3 years old are also removed as they have not enough historical records to indicate their physical condition until the selected target time. There are 462,809 patients retained after the steps outlined above.

### Statistical analysis

In the following analysis, we randomly select 70% of the patients for training the logistic regression model and retain 30% for testing the performance of the trained model. The model predicts admission at the target year for each patient.

First, we present the descriptive characteristics of key factors. A Chi-square test is implemented to compare the frequency of various categories for categorical variables in the admitted and non-admitted cohorts. For continuous variables, a t-test is used to accept or refute the null hypothesis that the corresponding means of the variables are equal in the admitted and non-admitted cohorts.

Second, following Brisimi et al. [22], we utilize a statistical hypothesis test comparing the sample difference of proportions between the insured and uninsured patients. The methodology of this method can be found in [23]. With this method, we split our data set into two groups of size *N*_1_ and *N*_2_, respectively, where the first group includes insured patients and the second group the uninsured. Correspondingly, the admission rates are denoted by *p*_1_ and *p*_2_. Suppose that whether or not a patient has been insured does not influence their hospitalization. Then, *p*_1_ should be statistically similar to *p*_2_. Under the null hypothesis that *p*_1_=*p*_2_, the difference between *p*_1_ and *p*_2_ approximately complies with a normal distribution, whose mean *μ* and deviation *σ* equal to 0 and *PQ*(1/*N*_1_+1/*N*_2_), respectively, where *P*=(*p*_1_*N*_1_+*p*_2_*N*_2_)/(*N*_1_+*N*_2_) and *Q*=1−*p*. We can then use the estimator *z*=(*p*_1_−*p*_2_)/*σ* to assess whether the null hypothesis holds or not.

Then, we conduct a logistic regression to further elaborate how a patient’s hospitalization was affected. In order to adjust the effect of confounding factors, we stratify the samples before analysis. More detailed methodology on the stratification technology can be found in Kleinbaum et al. [24]. Generally speaking, the distributions of cases and controls in different strata are usually substantially different. The stratification in our study is based on a three-dimensional tuple consisting of *Age*, *ER* *Visits*, and *Prior* *Admissions*. Appendix C presents the relation between admission and these tuple components. Even though lab tests are direct quantitative indicators deciding whether a patient should be hospitalized, we exclude these factors due to the serious deficiencies of lab test values and the difference in examination items, and use a three-dimensional tuple consisting of *Age*, *ER* *Visits*, and *Prior* *Admissions* as an indicator of severity. In our dataset, there are 72 different age-severity strata. The smallest and largest sample size in each stratum is 23 and 64,324, respectively.

The regression model is

$$\begin{array}{@{}rcl@{}} log(\frac{Prob(Admission)}{1-Prob(Admission)})&=&\beta_{0}+\beta_{1}Sex+\beta_{2}Race\\&&+\beta_{3}Marital\\ & &+\beta_{4}PrscNum +\beta_{5}Chronic\\&&+\beta_{6}Diagnosis\\ & &+ \beta_{7}Combinations \\&&+\beta_{8}Payer+\beta_{9}Severity \end{array} $$

where *Prob*(*Admission*) denotes the probability of hospitalization in the target time, ***β***=(*β*_0_,*β*_1_,...,*β*_9_) are unstandardized coefficients.

Finally, a Receiver Operating Characteristic (ROC) curve, which plots the sensitivity (or detection rate, or recall) as a function of the false positive rate (equal to one minus the specificity) is presented to demonstrate the performance of the model.

## Results

After the data pre-processing, our study population consisted of 462,809 patients, 67,332 of whom were admitted during the target year. Table 2 presents summary statistics of our sample. Patients who have been admitted are more likely to be male, white, and be divorced or separated. The number of patients insured by a government program is much higher than those with other payer status in our sample, which is consistent with a safety-net health care provider. This is mainly the result of the mission of safety-net hospitals to provide healthcare to all populations regardless of their payer status or ability to pay. The severity of illness, as defined earlier, is significantly different between admitted and non-admitted. Appendix C in Additional file 1 illustrates how the three factors we used to define severity affect admissions.

As outlined in Methods, we first use a hypothesis test to compare admission rates between the insured patients and the uninsured. In the hypothesis test, we obtain *z* = 49.3, which means that the probability that *p*_1_=*p*_2_ is much smaller than 0.0001. Therefore, we can reject the null hypothesis and assert that insurance status affects hospitalizations.

Then, we examine how payer status affects admissions with a conditional logistic regression model. Table 3 presents our results. We use Private as the reference payer status. The significantly positive unstandardized coefficients of Multiple Payers and Government indicate that the admission probability for patients who are totally or partially insured by government, controlling for other variables in the model, is higher than that for patients who are insured by private insures. Similarly, the significantly negative unstandardized coefficient of Uninsured indicates that the uninsured patients are less likely to be admitted than patients insured by private insurers.

**Table 3:** Impact of payers status on patients’ hospitalization

Variables	*β*^1^	*β*^∗^^2^	odds ratio^3^	*P*-value
*Payer*				
Multiple payers	0.671	0.153	(1.846, 2.072)	0.000
Government	0.194	0.097	(1.176, 1.253)	0.000
Other Payers	0.503	0.091	(1.496, 1.829)	0.000
Uninsured	-0.204	-0.044	(0.744, 0.893)	0.000
*Sex*				
Female	-0.286	-0.143	(0.731, 0.773)	0.000
*Race*				
Asian	-0.207	-0.045	(0.754, 0.876)	0.000
Hispanic	0.021	0.008	(0.983, 1.061)	0.290
Other Race	-0.312	-0.081	(0.690, 0.756)	0.000
Unknown Race	-1.182	-0.286	(0.273, 0.345)	0.000
White	0.315	0.149	(1.324, 1.418)	0.000
*Marital*				
Divorced	0.162	0.028	(1.099, 1.257)	0.000
Other Marital	-0.281	-0.059	(0.694, 0.822)	0.000
Seperated	0.044	0.005	(0.948, 1.151)	0.000
Single	-0.077	-0.037	(0.895, 0.957)	0.000
Unknown Marital	-0.932	-0.032	(0.151, 1.030)	0.000
Widow	0.338	0.049	(1.296, 1.516)	0.000
*PrscNum*	-0.199	-0.309	(0.813, 0.827)	0.000
*Chronic*	-0.589	-0.194	(0.527, 0.584)	0.000
*Combinations*	0.337	1.113	(1.395, 1.408)	0.000
*Diagnosis*				
Circulatory	0.543	0.124	(1.457, 2.036)	0.000
Congential Anomalies	-0.654	-0.036	(0.392, 0.689)	0.000
Digestive	0.198	0.052	(1.036, 1.436)	0.018
Endocrine	-1.080	0.182	(0.284, 0.406)	0.000
Genitourinary	-0.586	-0.132	(0.471, 0.659)	0.000
Illdefined	-1.356	-0.426	(0.218, 0.305)	0.000
Infections	-0.455	-0.079	(0.533, 0.755)	0.000
Injury & Poison	0.835	0.245	(1.958, 2.713)	0.000
Mental	-1.835	-0.431	(0.133, 0.191)	0.000
Metabolic & Immune	-0.294	-0.036	(0.624, 0.890)	0.001
Musculoskeletal	-1.081	-0.324	(0.288, 0.400)	0.000
Neoplasms	0.244	0.044	(1.079, 1.511)	0.005
Nutrition	-1.767	-0.059	(0.097, 0.300)	0.000
Pregnancy	2.581	0.477	(11.19, 15.58)	0.000
Respiratory	0.236	0.045	(1.069, 1.498)	0.006
Secondary	0.034	0.003	(0.842, 1.270)	0.747
Skin	-0.561	-0.115	(0.480, 0.678)	0.000
Supplementary1	-2.346	-0.745	(0.080, 0.114)	0.000
Unknown Diagnosis	-2.928	-0.473	(0.037, 0.076)	0.000

^1^*β* corresponds to the unstandardized coefficients from the regression model.

^2^*β*^∗^ corresponds to the standardized coefficients from the regression model. *β*^∗^ is calculated with the method suggested by Agresti[26] and Menard[30]: *β*^∗^= *βS*_*x*_, where *S*_*x*_ is the standard deviation of predictor *x*.

^3^The 5th column presents the 95% confidence interval of odds ratio

The specific influence of payer status on admission can be further elaborated by the corresponding odds ratios. (1) The odds ratio of Multiple Payers is 1.956, indicating that the odds of being admitted increase by 95.6% when the variable Multiple Payers increases. (2) The odds ratio of Government (odds ratio = 1.214) indicates that the odds of being admitted for patients who are insured by the government is 21.4% higher than the rest of the population. (3) The odds ratio of Uninsured (odds ratio = 0.815) indicates that uninsured patients are less likely to be admitted than insured patients when controlling for other variables.

The standardized coefficients in the 5th column in Table 3 indicate how many standard deviations of change in logit(*Admissions*) are associated with one standard deviation increase in the independent variables. According to Menard [25] and Agresti [26], standardized coefficients can be used to compare the relative influence of independent variables within a logistic regression model when the independent variables are measured in different units of measurements. Similar application of standardized coefficients can be found in [27]. In our findings, *Combinations* has the greatest influence in hospitalization among other factors (See Appendix B in Additional file 1 for the graph of ordered standardized coefficients). As the standardized coefficients value shown in Table 3, for example, a unit increase in *Combinations* is associated with a 1.113 increase in hospitalization. The values for payer status are somewhat lower (for example, 0.153 for Multiple payers, 0.097 for Government). On the other hand, the standardized coefficient values reflect that Multiple payers has the greatest impact on admissions among all payer status, successively followed by Government, Private and Uninsured.

### Validity check

**Fig. 2:**
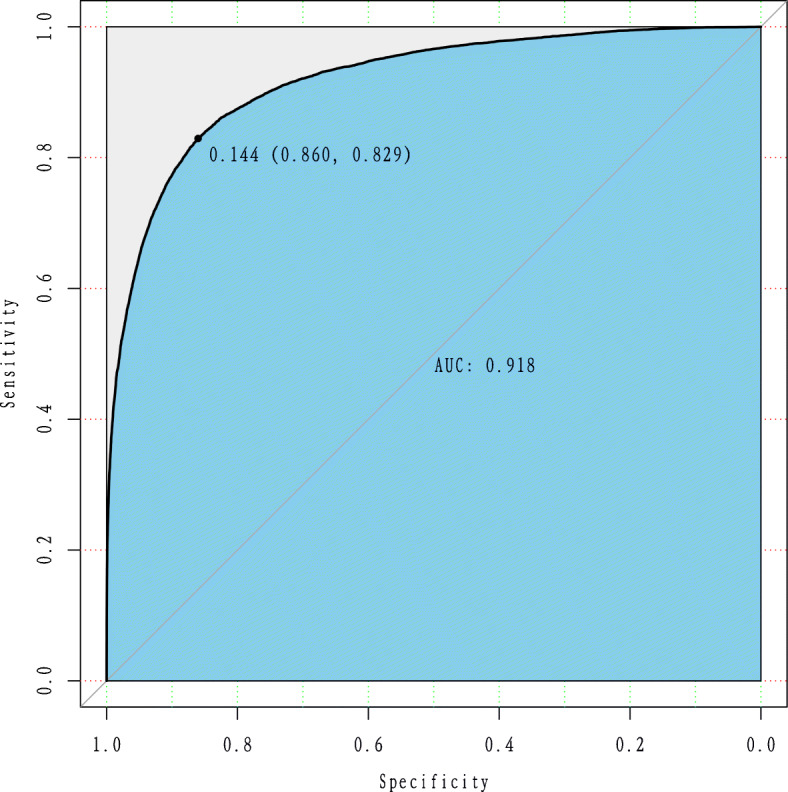
ROC curve to reveal the model’s prediction performance

We validate the estimated model via its prediction accuracy on a dataset distinct from the one used for training. Figure 2 plots the ROC curve for a random split of the data into a training and test set. The model has an Area Under the ROC Curve (AUC) of 92%, indicating excellent predictive power.

## Discussion

In an analysis of the relationship between admissions and payer status in a safety-net hospital, we found that uninsured patients are still less likely to be admitted than insured patients after controlling for demographics and prior medical conditions. These results are consistent with a streamline of work demonstrating inequity in access to health care[10, 13]. Generally, most uninsured people are in low-income families and may not be receiving public financial assistance for various reasons (e.g., age, income cutoff of financial assistance, undocumented immigrants, mental illness). As a consequence, it is also possible that they may not seek timely health care or receive treatment due to their economic, legal, or mental illness condition.

Further, we also found that patients who are partially or totally insured by government are more likely to be admitted than those who are insured by private insurers. Though we have controlled for prior medical conditions and age, still the association is significant. According to Exhibit A1 in the Additional file 1, among patients insured by government, 77.18% are totally or partially covered by Medicaid, therefore, we can infer that the high odds ratio of admission for patients insured by government are mainly driven by patients with Medicaid. Since our findings are not causal, this relationship only demonstrates an association between payer status and admissions. There may be several plausible explanations for this association:

(1) Low-income patients tend to experience worse health quality and delayed diagnosis and treatment, leading to worse health conditions when they are forced to seek health care. This is also consistent with Adepoju et al. [8] and Giacovelli et.[3]. From this perspective, there may be more opportunities to design preventive care programs for patients that are insured by the government, compared to those insured by private insurers, for basic preventative care can significantly decrease avoidable hospitalizations [28]. Given the characteristics of the safety net hospital, we feel this explanation is very plausible.

(2) Patients with enough ability to pay may elect to seek care at a non-safety-net hospital setting, and these admissions are not present in our dataset. As suggested by Sutton et al. [17], inpatients in a safety-net hospital are usually associated with pregnancy and injuries, whereas inpatients in non-safety-net hospitals are more likely to be related to surgery. (3) Patients insured by Medicare and Medicaid include patients with disabilities who may require more frequent hospitalizations. However, this effect is mitigated by the fact that our model controls for the complexity of a patient’s health condition.

Our results also highlight additional information on factors that can influence a patient’s hospitalization. Demographics play a role in explaining the relationship between admissions and payer status. Among all factors included in our analysis, the number of other diseases for a patient (combinations) contributes the most to a hospitalization since this variable has the largest standardized coefficient. In addition, the standardized coefficients suggest that demographics are not a major factor.

There exist significant differences among disease types in admissions. Specifically, the top 3 disease types contributing to admission are Pregnancy, Injury & Poison and Endocrine (see Table 3 or Appendix B in Additional file 1).

Our results can lead to two policy recommendations. (1) Attention is needed on improving the access to care for vulnerable (low-income) patients, for example, by actively advertising free care programs, reaching out to community organizations with better access to these individuals, or offering assurances that access to care is not linked to immigration procedures. (2) In order to reduce preventable admissions, basic preventive care services should be enhanced. The policy recommendations are in line with the World Health Organization’s demand for developing long-term growth in health spending and effective health policies. Similar policy recommendations can be found in Jakovljevic et al. [29], where policies on improve the low-income patients health by promoting patients’ healthy lifestyle and enhancing basic care are called on.

### Limitations

First, the differences among payer groups may be biased by not controlling for lab tests and other unaccounted factors in our analysis, including disability status for those insured by government programs. Though we use surrogates for illness severity, they may not fully account for the true health status of a patient.

Second, the identification of hospitalization is imprecise. Hospitalized patients in our sample are limited to those who were actually hospitalized, omitting those who were suggested to be hospitalized but did not follow through, or elected to be hospitalized at a different hospital, or even moved or died. This type of label bias makes our results underestimate the probability of hospitalization.

Third, the lack of patient source. Due to the lack of information on whether a patient has primary care though the hospital we considered, we cannot separate patients who get their primary care and those who come just for a hospitalization. This may lead to deficiency in historical information for some admitted patients.

Fourth, some estimates of independent variable coefficients may not be accurate, in part due to the dependence between payer status and disease types. Nevertheless, according to a rough rule in estimating the severity of collinearity resulting from dependence between variables [30, 31], the collinearity can be tolerated if the standardized coefficient is smaller than 1 or the unstandardized coefficient is smaller than 2. In our analysis, the standardized coefficients are all smaller than 1 except for Combinations, which suggests that the results are plausible.

## Conclusions

This study provides a snapshot of the differences of hospitalization for patients with different payer status. and it is a first such study done at a facility level.

Based on the insurance status, we stratified patients into five groups: government-sponsored, private-sponsored, multiple-sponsored, uninsured and other. We then used a conditional logistic regression model that is able to control for the influence of a patient’s illness severity to investigate the influence of other potential social factors. We found that coverage by Medicaid or Medicare plays a significant role in improving access to care (e.g., hospitalization) for low-income patients, but there might exist preventable admissions for this group of patients. For uninsured patients, inequity in terms of hospitalization still exists. Therefore, strategies to prevent hospitalizations for low-income insured patients and providing help for uninsured patients may be advisable.

We believe this study offers some insights for hospital administrators and policy makers on disparity of payer status on hospitalization. The findings can serve as a baseline from which to measure the anticipated effect of measures to reduce disparity of payer status in hospitalization.

## Supplementary Information

**Additional file 1** Appendix. This file contains the following content: (1)Summary characteristics of Payers; (2)The order of importance on influencing hospital admission; (3)Relationship between admissions and severity representatives; (4)The descriptive characters of variables.

## Data Availability

The data that support the findings of this study are available from Boston Medical Center in Massachusetts but restrictions apply to the availability of these data, which were used under license for the current study, and so are not publicly available. Data are however available from the authors upon reasonable request and with permission of the Medical Center.

## Abbreviations

ICD9: International classification of diseases 9th version
HIV: human immunodeficiency virus
ER: emergency room
ROC curve: Receiver Operating Characteristic curve
