# Supplementary material for: The impact of payer status on hospital admissions: evidence from an academic medical center
Source: BMC Health Serv Res. 2021 Sep 7;21:930. doi: 10.1186/s12913-021-06886-3 (PMC8425077; doi:10.1186/s12913-021-06886-3)
Supplement: Supplementary file 1 — Additional file 1 Appendix. This file contains the following content: (1)Summary characteristics of Payers; (2)The order of importance on influencing hospital admission; (3)Relationship between admissions and severity representatives; (4)The descriptive characters of variables. [file 12913_2021_6886_MOESM1_ESM.docx]

**Appendix A. Summary characteristics of Payers**

| **Payer status** | **Payer(s)** | **# of patients** | **Percentage** |
| --- | --- | --- | --- |
| Multiple payers (*N*=25,646) | Government + Private | 25,649 | 100% |
| Government (*N*=245,178) | Medicare | 55,954 | 22.82% |
|  | Medicaid | 162,691 | 66.36% |
|  | Medicare + Medicaid | 26,533 | 10.82% |
| Private (*N*=15,670) | Commercial | 147,991 | 96.19% |
|  | Accident | 5,838 | 3.79% |
|  | Commercial + Accident | 17 | 0.01% |
| Uninsured (*N*=22469) | Self-pay | 7,205 | 32.07% |
|  | Unknown | 15,264 | 67.93% |
| Other (*N*=15,670) | Other | 15,670 | 100% |

Note: Health safety net is a program provided by acute care hospitals or community health centers, which is available to uninsured or underinsured patients. Commonwealth care is a supplemental program in Massachusetts for patients who have both Medicare and Medicaid. Thus, patients with Medicaid include those who are insured by Medicaid, Health safety net, or both Medicaid and Health safety net; Patients with Medicare + Medicaid include those who are insured by Commonwealth care, or both Medicare and Medicaid. In addition, uninsured patients consist those who are labeled as self-pay or whose payer information is missing.

## Exhibit A1. Summary characteristics of Payers

Since our data comes from a safety net hospital, patients insured by government, either totally or partially, constitute 58.52% of all patients. For patients totally insured by government, 66.36% are covered by Medicaid, indicating that there is a large proportion of low-income patients in our dataset. It is worth noting that there exists a substantial number of patients lacking information on their payer status in the group of uninsured patients, which may overrate the proportion of uninsured patients.

**Appendix B. The order of importance on admission**

**
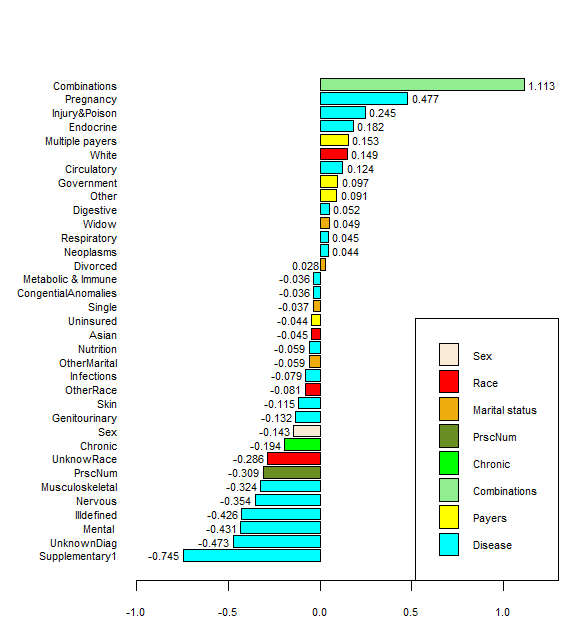
**

**Figure A1. Ordered standardized coefficients.**

The standardized coefficients compare the relative importance of different variables which may have different units and impact on admissions. From Figure A1, it can be seen that the most important factor affecting hospitalization is *Combinations*, which we defined as the number of diagnoses associated with a patient other than the primary admitting diagnosis. The top four diseases with a positive effect on hospitalizations are Pregnancy, Injury & Poison, Endocrine (e.g., diabetes mellitus), and Circulatory. Comparing to the above disease types and severity of a patient’s health condition, being insured has a weaker but positive effect on hospitalization. Demographics are the least important factors. Providing patients with prescriptions plays a significant role in reducing the logit of hospitalization.

**Appendix C. Relationship between admissions and tuple components**

The relationship is revealed with a logistic regression:

$$\log\left( \frac{Prob(Admission)}{1-Prob\left( Admission \right)} \right)=Intercept+\beta_{1}*Age+\beta_{2}*ER visits+\beta_{3}*Prior Admissions$$

The regression results are presented in the following table.

## Exhibit A2. Relationship between admissions and tuple components

| **Variables** | ***β*** | ***β**** | **Odds ratio** | **Significance** |
| --- | --- | --- | --- | --- |
| *Age* |  |  |  |  |
| [10,20) | 0.589 | 0.183 | 1.802 | 0.000 |
| [20,30) | 0.940 | 0.386 | 2.560 | 0.000 |
| [30,40) | 0.968 | 0.363 | 2.633 | 0.000 |
| [40,50) | 0.936 | 0.339 | 2.550 | 0.000 |
| [50,60) | 1.061 | 0.370 | 2.889 | 0.000 |
| [60,70) | 1.214 | 0.350 | 3.459 | 0.000 |
| 70 or older | 1.600 | 0.418 | 4.953 | 0.000 |
| *ER visits* |  |  |  | 0.000 |
| = 1 ER visit record | -1.026 | -0.394 | 0.358 | 0.000 |
| > 1 ER visit records | -0.546 | -0.133 | 0.580 | 0.000 |
| *Prior Admissions* |  |  |  | 0.000 |
| = 1 prior admission record | 3.796 | 0.632 | 44.522 | 0.000 |
| > 1 prior admissions records | 5.009 | 0.793 | 149.755 | 0.000 |

Note: *β* denotes the unstandardized coefficients; *β^*^* denotes the standardized coefficients. The standardized coefficients are calculated with the method suggested by Agresti^21^ and Menard^22^: *β^∗^=βS_x_*, where *S_x_* is the standard deviation of predictor *x*. The reference levels are age of [0,10), 0 ER visit records and 0 prior admission record for the three categorical variables, respectively. *R*^2^=0.227.

Earlier admission records are highly correlated with future admissions. Patients who have had emergency room visit records are less likely to be admitted. It can be argued that patients who visit the emergency room may address their ailment and reduce their likelihood of a future hospitalization. Based on the scale of the coefficients, we find that the most important factor influencing a patient's hospitalization is the number of prior admissions.

**Appendix D. The descriptive characters of variables**

We summarized the descriptive characters of related variables in this section. For each continuous variable, we calculated its mean and standard deviation; For each categorical variable, we present the percentage of each level.

## Exhibit A3. Descriptive characters of continuous variables.

| **Continuous variables** | **Payer** | | | | |
| --- | --- | --- | --- | --- | --- |
|  | Multiple payers | Government | Private | Uninsured | Other payers |
| *Age* (years) | 40.5 | 42.2 | 37.5 | 34.2 | 39.3 |
|  | (21.8) | (20.1) | (16.4) | (15.7) | (18.4) |
| *PrscNum* | 0.572 | 0.48 | 0.329 | 0.13 | 0.182 |
|  | (2.08) | (1.76) | (1.19) | (0.844) | (0.712) |
| *Combinations* | 2.91 | 3.44 | 2.24 | 1.44 | 0.0874 |
|  | (3.26) | (3.63) | (2.72) | (1.99) | (1.88) |
| *ER visits* | 0.476 | 0.385 | 0.266 | 0.711 | 0.077 |
|  | (1.472) | (1.747) | (0.356) | (0.485) | (0.174) |
| *Prior admissions* | 0.162 | 0.195 | 0.052 | 0.024 | 0.032 |
|  | (0.785) | (1.219) | (0.188) | (0.106) | (0.124) |

Note: The number in each parenthesis denotes the standard deviation. *Age* in the above table is measured in years.

## Exhibit A4. Descriptive characters of diagnosis.

| **Diagnosis** | **Payer** | | | | |
| --- | --- | --- | --- | --- | --- |
|  | Multiple payers | Government | Private | Uninsured | Other payers |
| Pregnancy | 10.13% | 4.33% | 1.78% | 1.26% | 1.56% |
| Injury & Poison | 6.59% | 7.48% | 12.78% | 17.10% | 3.55% |
| Endocrine | 2.64% | 3.22% | 3.00% | 1.53% | 0.52% |
| Circulatory | 6.16% | 6.46% | 4.42% | 3.15% | 1.81% |
| Digestive | 7.68% | 8.25% | 5.97% | 10.12% | 2.87% |
| Respiratory | 3.64% | 4.02% | 3.65% | 4.97% | 1.09% |
| Neoplasms | 3.13% | 3.64% | 3.78% | 0.84% | 1.16% |
| Blood | 0.52% | 0.50% | 0.39% | 0.34% | 0.17% |
| Metabolic & Immune | 0.94% | 1.64% | 1.65% | 0.74% | 0.22% |
| Congenital Anomalies | 0.23% | 0.34% | 0.31% | 0.20% | 0.06% |
| Nutrition | 0.09% | 0.09% | 0.15% | 0.06% | 0.04% |
| Infections | 3.24% | 3.17% | 2.62% | 4.03% | 5.02% |
| Skin | 3.11% | 4.40% | 4.90% | 4.06% | 0.91% |
| Genitourinary | 4.40% | 5.35% | 5.97% | 5.45% | 1.56% |
| Musuloskeletal | 7.45% | 10.03% | 11.48% | 8.54% | 2.55% |
| Nervous | 9.00% | 7.86% | 6.15% | 4.45% | 2.25% |
| Ill-defined | 10.57% | 10.39% | 10.67% | 14.17% | 22.64% |
| Mental | 5.02% | 6.74% | 4.70% | 8.20% | 1.07% |
| Unknown Diagnosis | 2.74% | 2.11% | 1.97% | 0.28% | 22.08% |
| Supplementary1 | 12.05% | 9.27% | 13.07% | 10.22% | 28.67% |

**Exhibit A5. Descriptive characters of categorical variables except for diagnosis**

| **Categorical variables** | **Payer** | | | | |
| --- | --- | --- | --- | --- | --- |
|  | Multiple payers | Government | Private | Uninsured | Other payers |
| *Race* |  |  |  |  |  |
| Asian | 5.14% | 4.16% | 4.92% | 3.86% | 17.07% |
| Black | 36.33% | 31.52% | 22.48% | 37.68% | 24.01% |
| Hispanic | 28.44% | 23.86% | 10.55% | 19.81% | 12.13% |
| Other Race | 10.38% | 7.72% | 6.18% | 8.07% | 5.99% |
| Unknown Race | 5.55% | 5.31% | 7.64% | 5.55% | 7.38% |
| White | 14.16% | 27.43% | 48.23% | 25.03% | 33.42% |
| *Marital* |  |  |  |  |  |
| Divorced | 2.04% | 3.93% | 2.37% | 1.80% | 2.54% |
| Married | 21.75% | 20.66% | 30.75% | 15.62% | 25.77% |
| Other Marital | 4.53% | 4.52% | 4.63% | 3.92% | 6.58% |
| Separated | 1.55% | 1.98% | 0.93% | 1.21% | 0.74% |
| Single | 66.34% | 65.74% | 60.51% | 76.31% | 62.89% |
| Unknown Marital | 0.13% | 0.11% | 0.11% | 0.20% | 0.08% |
| Widow | 3.66% | 3.05% | 0.71% | 0.94% | 1.40% |
| *Sex* |  |  |  |  |  |
| Male | 39.90% | 45.65% | 48.15% | 59.61% | 62.57% |
| Female | 60.10% | 54.35% | 51.85% | 40.39% | 37.43% |
| *Chronic* | 14.20% | 14.10% | 9.63% | 5.36% | 22.20% |
| *Age(group)* |  |  |  |  |  |
| [0, 10) | 5.72% | 5.39% | 3.50% | 4.12% | 1.04% |
| [10, 20) | 15.53% | 9.67% | 11.28% | 13.36% | 12.60% |
| [20, 30) | 19.14% | 17.89% | 25.40% | 32.13% | 28.23% |
| [30, 40) | 16.40% | 15.93% | 18.17% | 19.18% | 16.97% |
| [40, 50) | 11.14% | 15.81% | 16.09% | 14.54% | 13.86% |
| [50, 60) | 7.63% | 14.47% | 15.90% | 9.61% | 10.67% |
| [60, 70) | 10.57% | 10.26% | 7.87% | 4.62% | 8.75% |
| 70 or older | 13.86% | 10.58% | 1.78% | 2.44% | 7.88% |
| *ER Visits* |  |  |  |  |  |
| = 0 ER visit record | 76.81% | 76.19% | 78.02% | 40.25% | 94.41% |
| = 1 ER visit record | 2.89% | 16.72% | 18.79% | 52.02% | 4.38% |
| >1 ER visit records | 20.31% | 7.08% | 3.19% | 7.73% | 1.21% |
| *Prior Admissions* |  |  |  |  |  |
| = 0 prior admission | 92.41% | 92.50% | 97.25% | 98.91% | 98.14% |
| = 1 prior admission | 4.16% | 3.71% | 1.79% | 0.69% | 1.26% |
| >1 prior admissions | 3.44% | 3.79% | 0.96% | 0.41% | 0.60% |

From the above tables, we can observe that there exists a difference in frequency among levels in each categorical variable, indicating that the payer and each categorical variable may be dependent to some extent. Serious dependence among variables results in collinearity, which will make the estimated coefficients inaccurate and unstable. However, absolute independence is rare. Hence, our objective is to avoid significant collinearity. According to the method in Menard^18^, a rough rule to detect collinearity in logistic regression is based on the standardized coefficients: if the standardized coefficients are smaller than 1, we can infer that there is no serious collinearity among variables. In our study, the standardized coefficient of *Combinations* is greater than 1, however, the excess is not that much (see Exhibit 4). From the application level, we can conclude that there is no substantial collinearity problem in our data set.
